# Recombinant Soluble Respiratory Syncytial Virus F Protein That Lacks Heptad Repeat B, Contains a GCN4 Trimerization Motif and Is Not Cleaved Displays Prefusion-Like Characteristics

**DOI:** 10.1371/journal.pone.0130829

**Published:** 2015-06-24

**Authors:** Ivy Widjaja, Alan Rigter, Shamir Jacobino, Frank J. M. van Kuppeveld, Kees Leenhouts, Concepción Palomo, Jose A. Melero, Jeanette H. W. Leusen, Bert Jan Haijema, Peter J. M. Rottier, Cornelis A. M. de Haan

**Affiliations:** 1 Virology Division, Department of Infectious Diseases & Immunology, Faculty of Veterinary Medicine, Utrecht University, 3894 CL, Utrecht, The Netherlands; 2 Mucosis B.V., Meditech Center, L.J. Zielstraweg 1, 9713 GX Groningen, The Netherlands; 3 Department of Immunology, University Medical Center Utrecht, 3508 AB, Utrecht, The Netherlands; 4 Centro Nacional de Microbiología and CIBERES, Instituto de Salud Carlos III, Majadahonda, 28220, Madrid, Spain; Deutsches Primatenzentrum GmbH - Leibniz-Institut fur Primatenforschung, GERMANY

## Abstract

The respiratory syncytial virus (RSV) fusion protein F is considered an attractive vaccine candidate especially in its prefusion conformation. We studied whether recombinant soluble RSV F proteins could be stabilized in a prefusion-like conformation by mutation of heptad repeat B (HRB). The results show that soluble, trimeric, non-cleaved RSV F protein, produced by expression of the furin cleavage site-mutated F ectodomain extended with a GCN4 trimerization sequence, is efficiently recognized by pre- as well as postfusion-specific antibodies. In contrast, a similar F protein completely lacking HRB displayed high reactivity with prefusion-specific antibodies recognizing antigenic site Ø, but did not expose postfusion-specific antigenic site I, in agreement with this protein maintaining a prefusion-like conformation. These features were dependent on the presence of the GCN4 trimerization domain. Absence of cleavage also contributed to binding of prefusion-specific antibodies. Similar antibody reactivity profiles were observed when the prefusion form of F was stabilized by the introduction of cysteine pairs in HRB. To study whether the inability to form the 6HB was responsible for the prefusion-like antibody reactivity profile, alanine mutations were introduced in HRB. Although introduction of alanine residues in HRB inhibited the formation of the 6HB, the exposure of postfusion-specific antigenic site I was not prevented. In conclusion, proteins that are not able to form the 6HB, due to mutation of HRB, may still display postfusion-specific antigenic site I. Replacement of HRB by the GCN4 trimerization domain in a non-cleaved soluble F protein resulted, however, in a protein with prefusion-like characteristics, suggesting that this HRB-lacking protein may represent a potential prefusion F protein subunit vaccine candidate.

## Introduction

Human respiratory syncytial virus (RSV) causes acute infections of the upper and lower respiratory tract. Symptoms of disease can be severe, especially in premature babies and in children with underlying health conditions; but also in the elderly, in adults with heart and lung disease and in immune-compromised individuals. Currently, the only available option to prevent RSV-mediated severe disease in premature infants is the administration of the RSV-neutralizing monoclonal antibody (MAb) Palivizumab (for recent reviews see [1,2]). A registered vaccine against RSV is not available.

**Fig 1:**
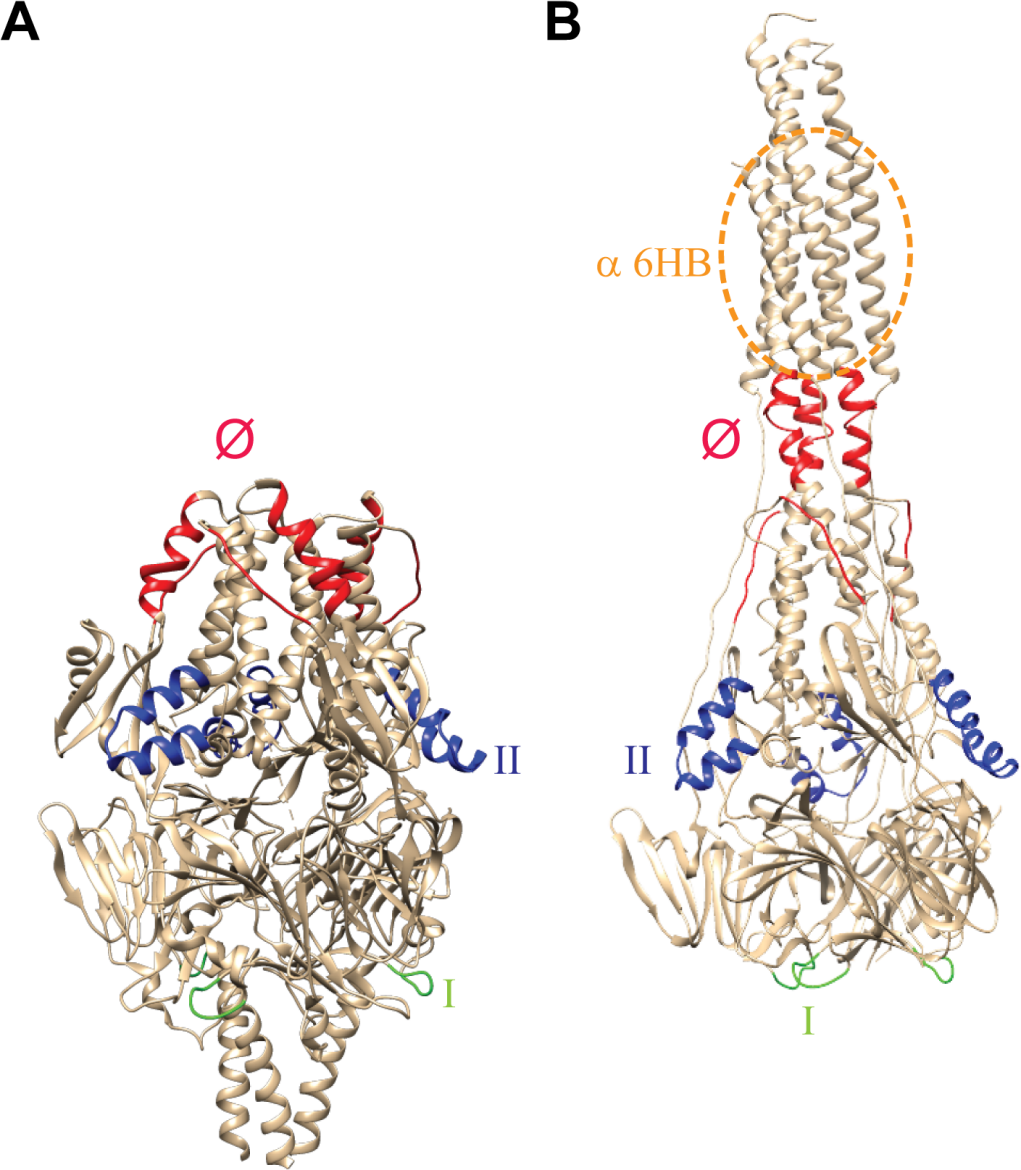
Overview of antigenic sites on pre- and postfusion F. (A) prefusion [8] and (B) postfusion [10] structures of RSV F. Antigenic sites recognized by antibodies used in this study are indicated (according to [8]): site I (green; recognized by MAb 131-2a), site II (blue; recognized by MAb Palivizumab), and site Ø (red; recognized by MAbs D25 and AM22). The region to which the α6HB PAb binds is indicated by the dotted orange circle. Site Ø is disrupted in postfusion F, while site I appears to be shielded in the prefusion conformation.

RSV is an enveloped, negative-strand RNA virus belonging to the genus Pneumovirus of the family *Paramyxoviridae*. Its envelope contains two major glycoproteins: the attachment protein G and the fusion protein F [1,3]. The RSV F protein, which forms homotrimers that radiate from the virion surface, is a class I fusion protein. Synthesized as a precursor (F0), the protein is cleaved by furin-like proteases into F2, p27 and F1 [4,5,6]. The F1 part contains heptad repeats A and B (HRA and HRB), the fusion peptide and the transmembrane domain. The F2 domain appears to be an important determinant of species tropism [7]. Trimers of disulfide-linked F1 and F2 form the metastable prefusion structure of F (Fig 1). Conformational changes in RSV F during membrane fusion ultimately result in the formation of the very stable postfusion structure that is characterized by the presence of a typical six-helix bundle (6HB) containing HRA and HRB in an antiparallel orientation [8,9,10] (Fig 1). In this latter conformation, the fusion peptide and the transmembrane domain are positioned in adjacent positions, upstream of HRA and downstream of HRB, respectively, consistent with the viral and host membranes being fused.

The RSV F protein appears very unstable in its native prefusion conformation. In contrast to other paramyxovirus F proteins, it does not require the G protein for fusion activation [3,11,12], while electron tomography studies suggest a large proportion of the F proteins present on cells and virions to be in a postfusion conformation [13]. Also recombinant soluble F protein ectodomains prefer to adopt a postfusion conformation, even when C-terminally extended with a trimerization domain [4,9,10,14,15,16]. Coexpression of such a recombinant protein with a prefusion-specific antibody was required to enable its crystallization in the prefusion conformation [8].

The RSV F protein, especially in its prefusion conformation, is considered an attractive vaccine candidate. Antibodies against prefusion F account for most of the RSV neutralizing activity found in human sera [17], while higher neutralizing titers were induced after immunization with pre- compared to postfusion F [18]. Although some neutralizing epitopes are preserved in the postfusion conformation [9,10], antibodies that recognize the prefusion-specific antigenic site Ø were shown to be much more potent in neutralizing RSV infection [8,14,19,20,21] than Palivizumab, which recognizes antigenic site II that is exposed on the surface of both the pre- and postfusion form of F.

In the present study, we studied whether recombinant soluble RSV F proteins could be stabilized in a prefusion-like conformation by mutation of heptad repeat B (HRB). Previously, we showed that recombinant soluble RSV F ectodomains extended with a trimerization domain and containing mutated furin cleavage sites (Flys-GCN) display high reactivity with prefusion-specific antibodies as compared to F proteins in the postfusion conformation [14]. We now show that Flys-GCN also displays antigenic site I that is recognized by the postfusion-specific antibody 131-2a. In addition, this protein was recognized by antibodies against the 6HB. We subsequently analyzed the effect of mutating HRB on the display of pre- and postfusion-specific antigenic sites. Our results indicate that HRB mutations that prevent the formation of the 6HB were not necessarily sufficient to inhibit display of postfusion-specific antigenic site I. However, complete deletion of HRB from non-cleaved, GCN4-extended F resulted in proteins with prefusion-like characteristics that no longer display the postfusion-specific antigenic site I.

## Results

### The poorly neutralizing MAb 131-2a is not able to recognize full length RSV F protein stabilized in the prefusion conformation

**Fig 2:**
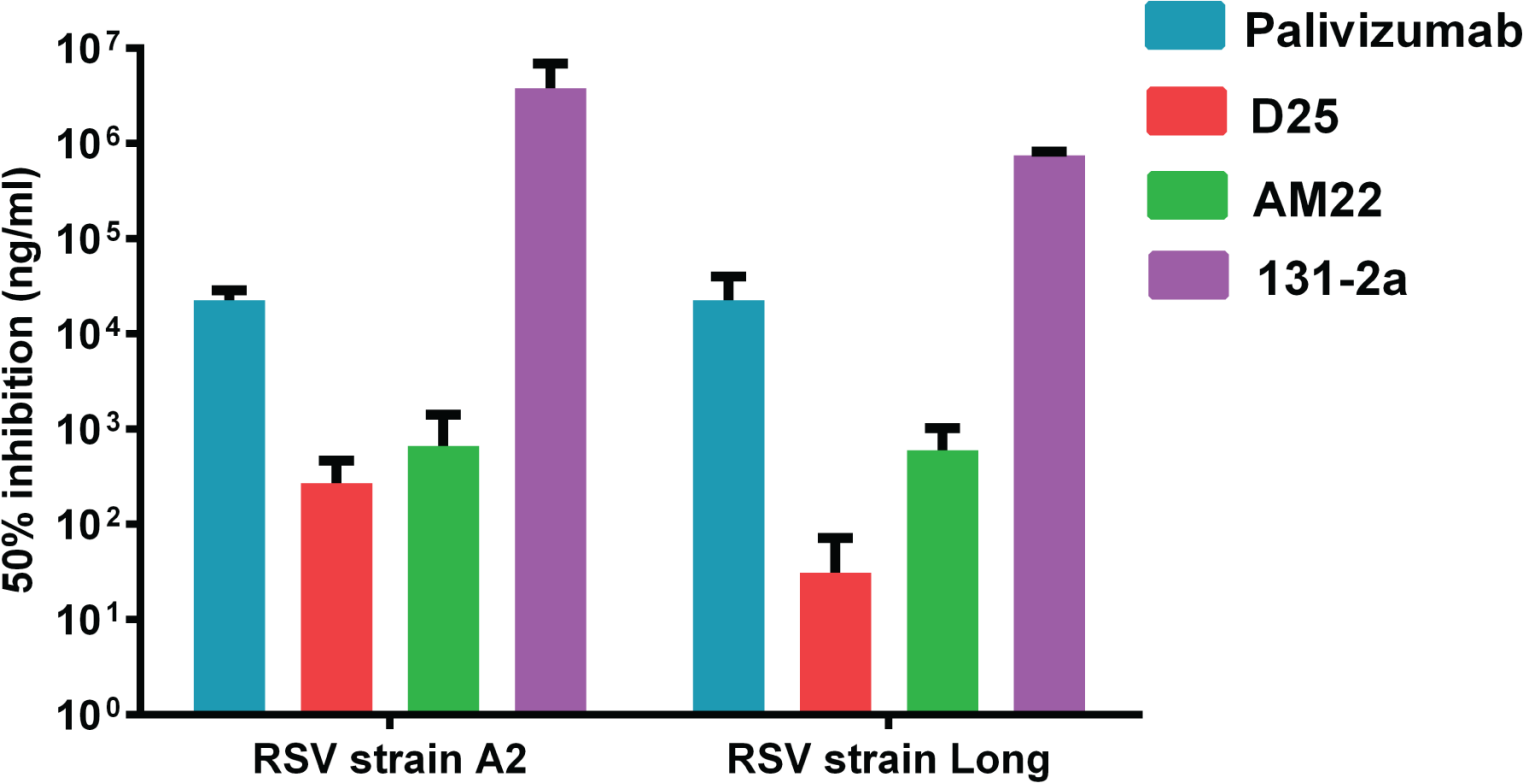
Neutralization of RSV by MAbs. The concentration needed of each MAb to achieve 50% neutralization of virus infectivity is graphed. Standard deviations are indicated.

In the present study we systematically analyzed the reactivity of different (conformation-specific) antibodies with recombinant RSV F proteins, with the aim of getting more insight into their antigenic structure. These antibodies include MAbs AM22 and D25 [8,19,20] (recognizing prefusion-specific antigenic site Ø), 131-2a (binding antigenic site I that has been proposed to be only available in the postfusion conformation [9]), Palivizumab (recognizing antigenic site II that is found on pre- and postfusion F), and a PAb directed against the 6HB (α6HB), which is only found in postfusion F [17,22] (Fig 1). As most of the MAbs were generated using a recombinant protein approach, we first validated their ability to neutralize RSV (Fig 2). D25 and AM22 (antigenic site Ø) displayed a higher neutralizing capacity than Palivizumab (antigenic site II). In addition, our results indicate that MAb 131-2a has only very limited virus neutralizing capacity.

**Fig 3:**
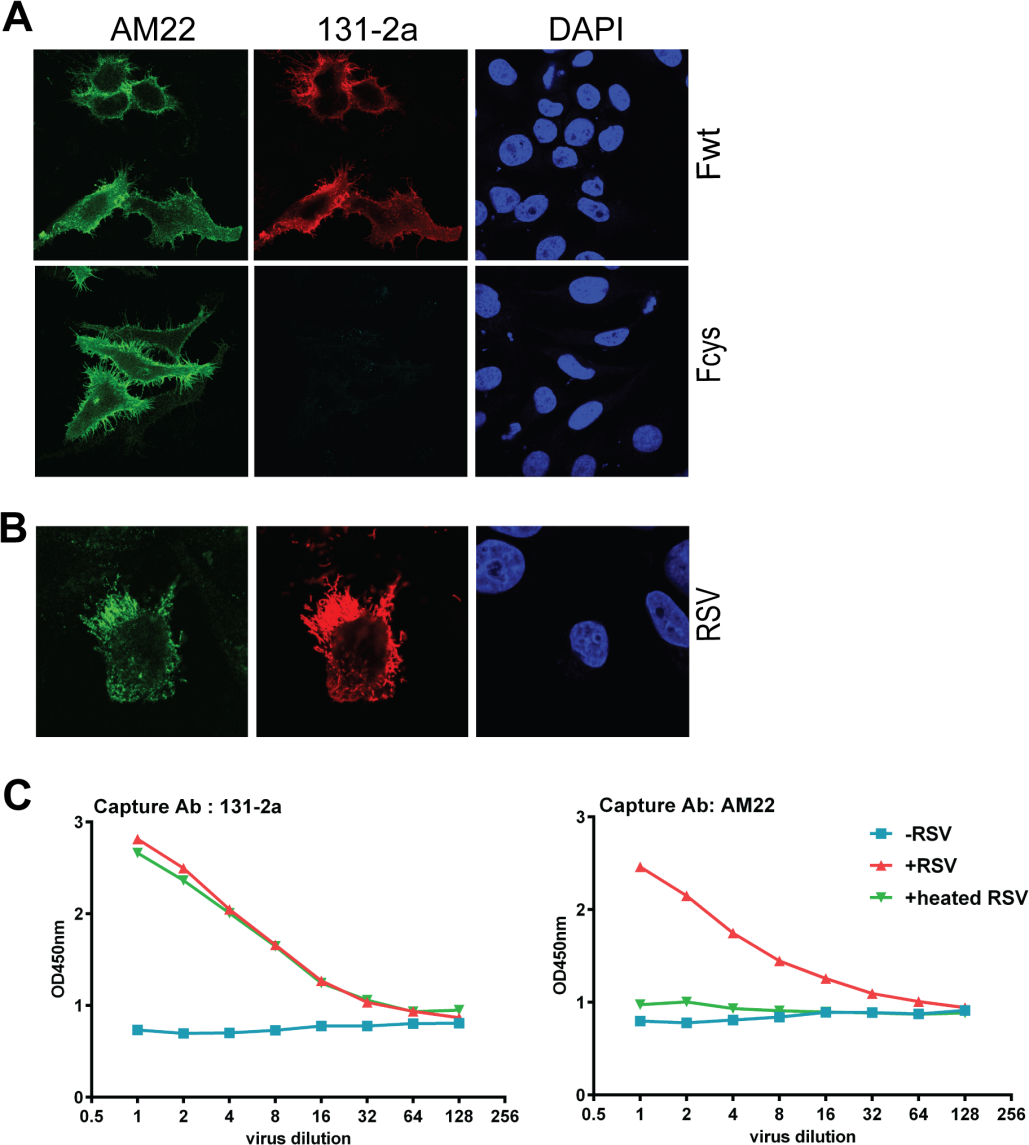
Reactivity of full length RSV F proteins with pre- and postfusion-specific antibodies. (A) Cells transfected with full length F protein expression plasmids or (B) infected with RSV (strain Long) were fixed and processed for immunofluorescence analysis as described in the Materials and Methods using MAbs AM22 and 131-2a. Nuclei were stained with DAPI. Wild type F protein (Fwt) or F proteins containing cysteine pairs in HRB (Fcys; [17]) were expressed. (C) Sandwich ELISA of RSV virus particles. RSV particles (+RSV) were captured using 131-2a or AM22. Capture of virus particles was detected using a PAb against RSV. As controls the experiment was performed without adding RSV particles (-RSV) or after heating of the particles (+heated RSV).

The poor neutralizing capacity of MAb 131-2a is in agreement with the assumption that this antibody predominantly recognizes the postfusion form of F [9]. Although recombinant F proteins that were efficiently recognized by prefusion-specific antibodies AM22 and D25 were in general not efficiently bound by 131-2a, some of these have been reported to display high reactivity with prefusion-specific antibodies as well as with 131-2a [18]. To establish that MAb 131-2a is indeed a postfusion-specific antibody that is not able to bind membrane-associated prefusion F we analyzed the ability of this antibody to recognize full-length RSV F stabilized in the prefusion conformation. To this end, cells were transfected with plasmids expressing the full length, wild-type F protein (Fwt) or a mutant version thereof, which contains cysteine residues in HRB (Fcys). These cysteine residues were previously reported to stabilize the prefusion-structure of RSV F by the formation of intersubunit disulfide bridges [17]. Cells expressing wild-type F were recognized by AM22 and 131-2a (Fig 3A and S1 Fig) as well as by D25 and Palivizumab (not shown). In contrast, cells expressing prefusion Fcys were bound by AM22 (and D25), but not by 131-2a. These results indicate that epitope I recognized by 131-2a is not exposed on prefusion F, while cell-associated wild-type F proteins may be recognized both by prefusion- and postfusion-specific antibodies.

The efficient binding of 131-2a to cells expressing RSV F may result somehow from the absence of other viral proteins such as G. To study this possibility, we also analyzed the reactivity of the MAbs with RSV-infected cells (Fig 3B). The results indicate that also infected cells display antigenic sites Ø (AM22) and I (131-2a). In agreement herewith, virus particles could be captured by antibodies recognizing either antigenic site Ø or I (Fig 3C). Heating of the virus particles abolished the reactivity of the particles with the prefusion-specific MAb AM22 but not with the postfusion-specific 131-2a. Similar results were obtained using a clinical isolate of RSV (unpublished results) or when captured particles were detected using pre- or postfusion-specific antibodies (S1 Fig). This latter result indicates that F proteins recognized by either pre- or postfusion-specific antibodies are present on the same particle. In summary, our results indicate that antibody AM22 (antigenic site Ø), but not the poorly neutralizing MAb 131-2a (antigenic site I) is able to bind the membrane-associated prefusion structure of RSV F. Antigenic site I is however readily exposed on virus- and cell-associated RSV F proteins.

### Non-cleaved, GCN4-extended RSV F ectodomain displays pre- and postfusion-specific epitopes

**Fig 4:**
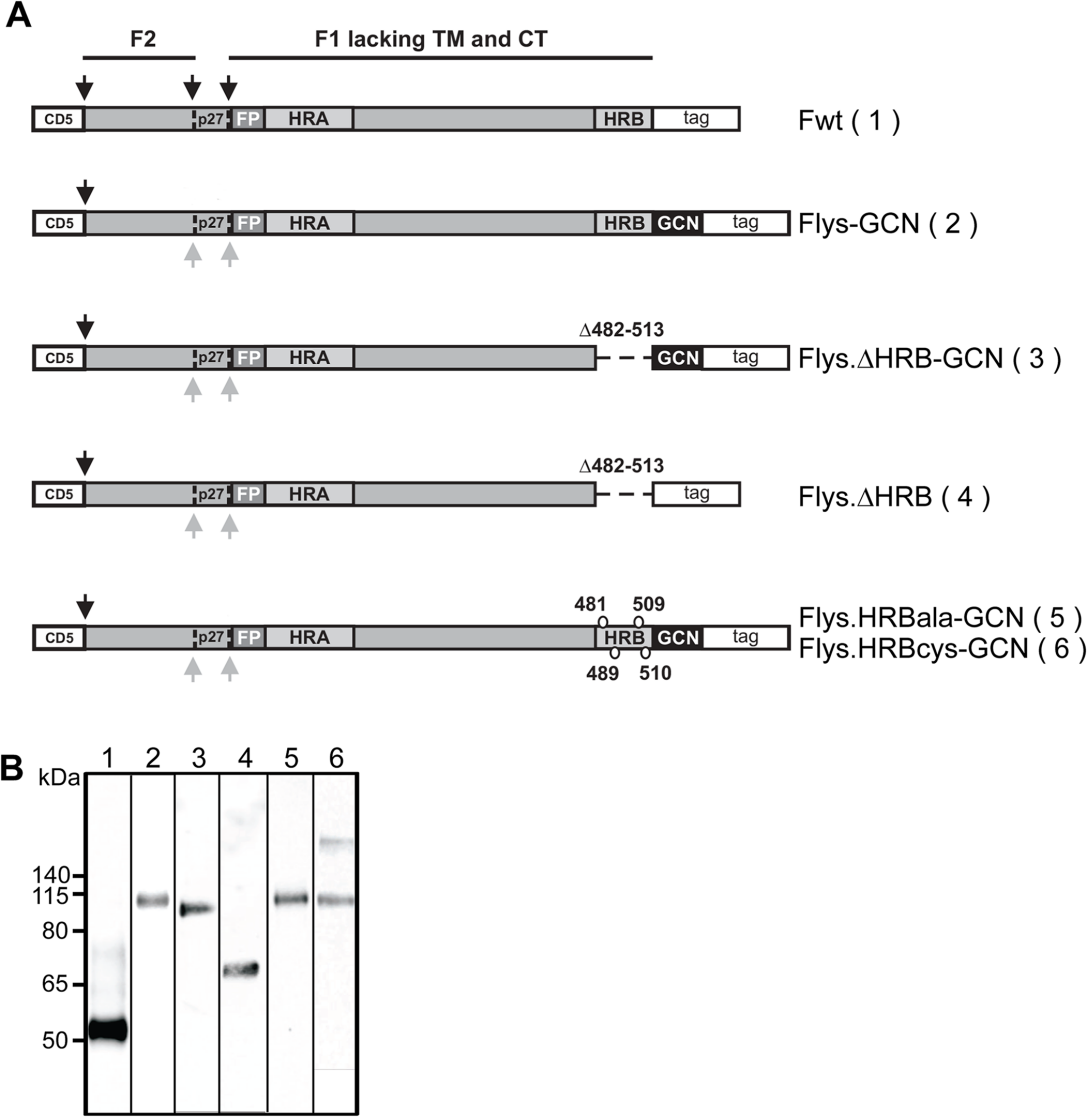
Schematic representation of the different recombinant soluble RSV F protein constructs. (A) RSV F ectodomains (amino acid 26–513) lacking the transmembrane domain (TM) and cytoplasmic tail (CT) were genetically fused to a CD5 signal peptide (CD5) and to a carboxy-terminal triple Strep tag II (tag). When indicated a GCN4 trimerization motif [34] and a LysM linker [35] immediately downstream of the RSV F ectodomain were added (indicated by GCN). The F2 and F1 subunits of F are indicated, as well as the p27 peptide (P27) that is released after furin cleavage. Protease cleavage sites are indicated by black arrows. Grey arrows indicate mutated furin cleavage sites. The approximate location of the fusion peptide (FP), heptad repeat A (HRA) and B (HRB) is also shown. The residues substituted in HRB by alanines or cysteines in Flys.HRBala-GCN and Flys.HRBcys-GCN, respectively, are indicated. The amino acids deleted in the F proteins that lack HRB are also indicated. (B) SDS-PAGE and Western blot analysis of recombinant soluble F proteins. Purified F proteins Fwt (lane 1), Flys-GCN (lane 2), Flys.∆HRB-GCN (lane 3), Flys.∆HRB (lane 4), Flys.HRBala-GCN (lane 5), Flys.HRBcys-GCN (lane 6) were analyzed by SDS-PAGE followed by Western blotting. Prior to SDS-PAGE analysis, the samples were resuspended in LSB containing 5% ME and heated at 96°C for 5 minutes. The size of the molecular mass markers (in kDa) is shown on the left side.

Next we analyzed the presence of pre- and postfusion-specific epitopes on recombinant soluble F proteins. The recombinant, furin-cleaved RSV F ectodomain (Fwt; Fig 4) was previously concluded to adopt the postfusion conformation as indicated by its low reactivity with prefusion-specific antibodies and the detection of SDS-resistant higher order structures by gel electrophoresis, which dissociate upon heating and which presumably correspond to the very stable 6HB-containing trimeric postfusion form of F [14]. Indeed, these higher order structures were recognized by antibodies against the 6HB (data not shown). Extension of the RSV F ectodomain with a GCN4 trimerization motif combined with mutation of the furin cleavage sites (Flys-GCN; Fig 4) resulted in F protein preparations in which the prefusion-specific antigenic site Ø was much better conserved with concomitant loss of the higher order structures [14]. Both the GCN4 domain and the absence of cleavage were shown to contribute to the increased reactivity of the F protein with the prefusion-specific antibodies[14].

**Fig 5:**
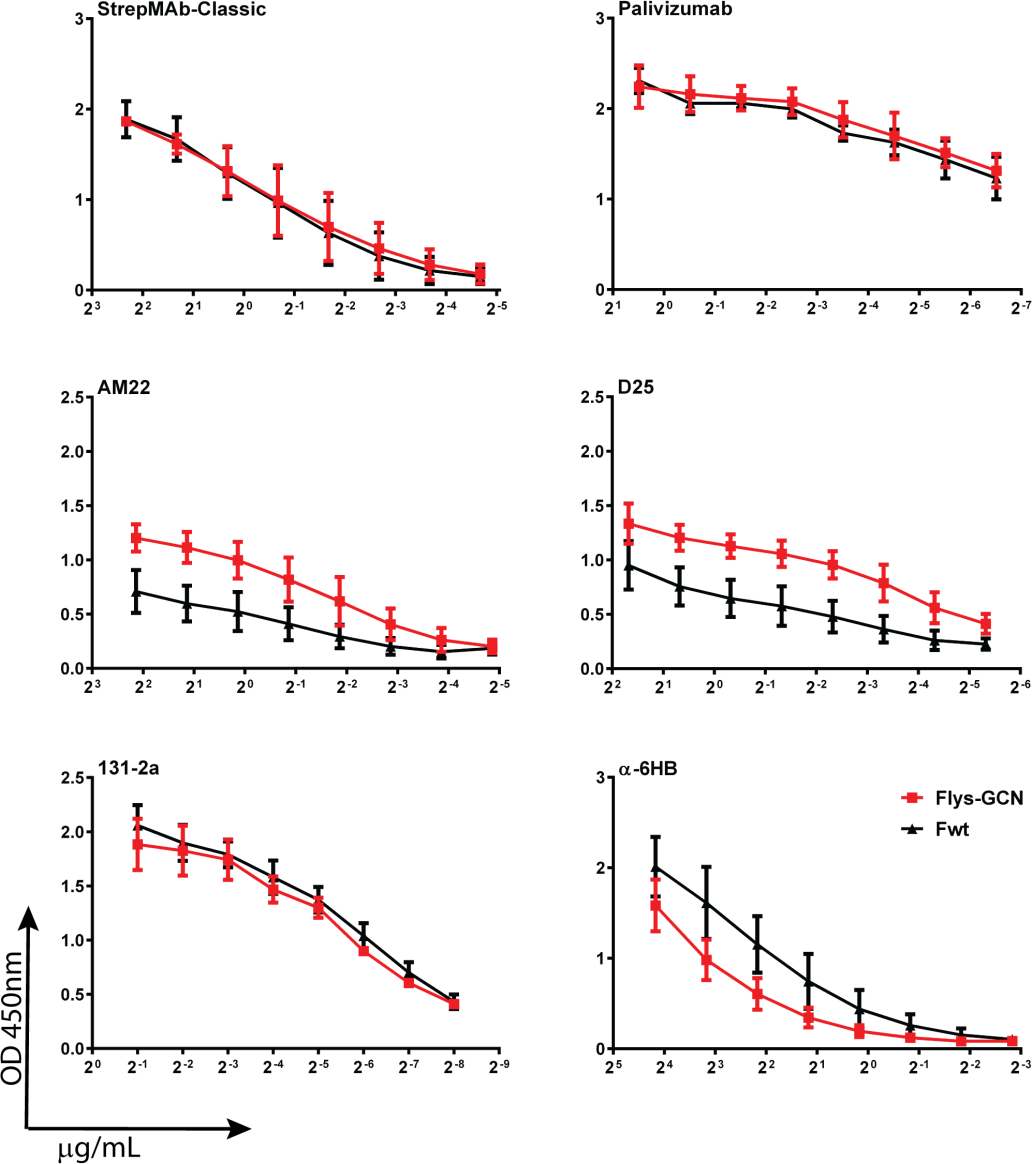
Antigenic analysis of Fwt and Flys-GCN. F protein preparations Fwt and Flys-GCN were coated in 96 well plates for ELISA analysis. The reactivity of the recombinant proteins with the different antibodies (indicated above each graph) was determined by applying 2-fold dilutions of these antibodies as indicated in the Materials and Methods. Binding of the antibodies was detected using HRP-conjugated secondary antibodies. The antibody dilution is indicated on the X-axes, while the Y-axes corresponds to optical density read at 450nm (OD450 nm). Error bars indicate standard deviations (n = 3).

We now studied the reactivity of these proteins (Fwt and Flys-GCN) with a much larger panel of antibodies, including the postfusion-specific antibodies 131-2a and α6HB by using an ELISA setup. Expression and purification of the recombinant soluble proteins was first analyzed by gel electrophoresis (Fig 4B). Flys-GCN displayed a lower gel electrophoretic mobility than Fwt, in agreement with previous results [14]. This lower molecular weight is explained by the cleavage of Fwt (therefore it lacks F2 and p27 with a predicted molecular weight of approximately 27 kDa) and Fwt not containing the GCN4 tag, which also includes the LysM domain (predicted combined molecular weight of GCN4 and LysM being approximately 21 kDa). The LysM domain enhances the expression and purification of GCN4-containing F protein ectodomains [14]. Coating of the ELISA plates with the recombinant proteins was checked with the MAb recognizing the Strep tag (StrepMAb classic), which resulted in very similar reactivity for both proteins (Fig 5 and S2 Fig). The two F proteins also displayed very similar reactivity with Palivizumab (antigenic site II), but differed in their reactivity with the antibodies AM22 and D25 that recognize antigenic site Ø, with the highest reactivity being observed for the Flys-GCN. Yet, the two proteins exhibited very similar reactivity with postfusion-specific antibody 131-2a (antigenic site I). Reactivity with PAb recognizing the 6HB (α6HB) was negatively affected by mutation of the furin cleavage sites and by the presence of the GCN4 domain. These results confirm that Fwt adopts the postfusion conformation and furthermore show that the Flys-GCN preparation exhibits prefusion-specific epitopes while it still displays reactivity with postfusion-specific antibodies.

### GCN4-extended F protein lacking HRB displays prefusion-like characteristics

We subsequently analyzed whether we could abrogate the reactivity of the recombinant soluble, non-cleaved, GCN4-extended F proteins with postfusion-specific antibodies by complete deletion of HRB (Flys.ΔHRB-GCN). As a control, the F protein lacking HRB was also expressed without being extended with the GCN4 trimerization domain (Flys.ΔHRB). Expression and purification of the recombinant soluble proteins was analyzed by gel electrophoresis (Fig 4B) and compared to the electrophoretic mobility of Fwt and Flys-GCN. Flys.ΔHRB-GCN and Flys-GCN displayed a similar gel electrophoretic mobility, which was larger than that of Fwt, in agreement with the latter protein being cleaved and not containing the GCN4 tag, which also includes the LysM domain [14]. Flys.ΔHRB ran at an intermediate position in agreement with this protein not being cleaved and lacking both HRB and the GCN4 extension.

**Fig 6:**
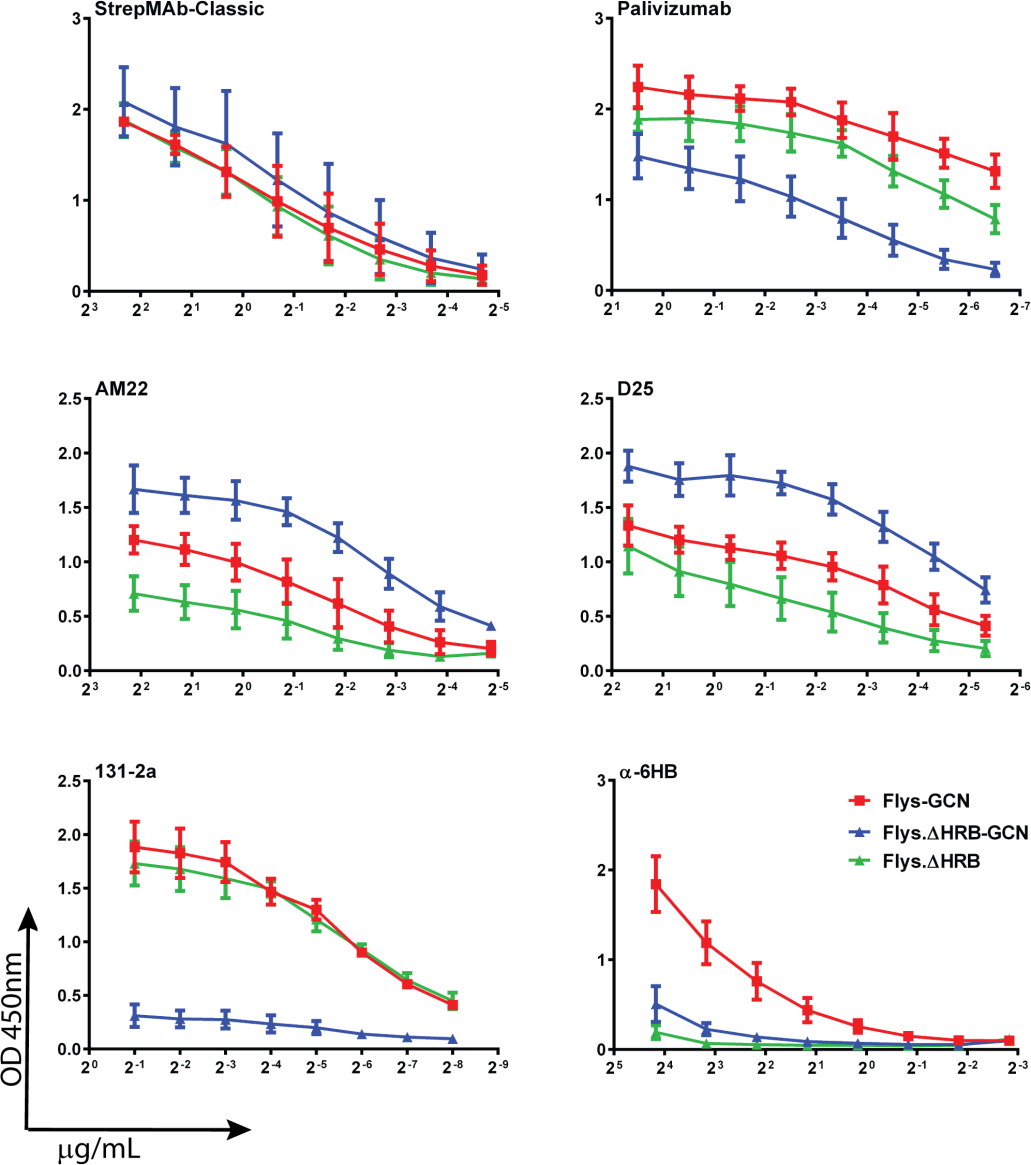
Antigenic analysis of F proteins lacking HRB. ELISA analysis of purified F proteins Flys-GCN, Flys.ΔHRB-GCN and Flys.∆HRB was performed similarly as described in the legend to Fig 5. Error bars indicate standard deviations (n = 3–5).

Subsequently, we analyzed the reactivity of the different recombinant soluble RSV F proteins with the panel of conformation-specific antibodies using the ELISA approach. Coating of the recombinant proteins was checked with the antibody recognizing the Strep tag, which resulted in very similar reactivity for Flys-GCN, Flys.ΔHRB-GCN and Flys.ΔHRB (Fig 6 and S3 Fig). The three F proteins were also bound by antibodies recognizing antigenic sites II (Palivizumab) and Ø (AM22 and D25), although consistent differences were observed between the different F protein preparations. Deletion of HRB from Flys-GCN decreased reactivity with the site II mAb (palivizumab) for an unknown reason. It is possible that placing the GCN4 trimerization domain next to the prefusion F protein head may contort its central region, thereby altering site II. Deletion of HRB from Flys-GCN increased reactivity with the site Ø MAbs (AM22 and D25) suggesting that removal of HRB from Flys-GCN stabilizes it in the prefusion form. In the absence of GCN4, the F protein lacking HRB displayed increased and decreased reactivity with antibodies recognizing antigenic site II (Palivizumab) and Ø (AM22 and D25), respectively, when compared to its GCN4-containing form. The different F proteins dramatically differed in their reactivity with the postfusion-specific antibodies 131-2a (antigenic site I) and α6HB. Flys-GCN was efficiently recognized by these latter two antibodies. The reactivity of the F protein lacking HRB with 131-2a completely depended on the absence or presence of the GCN4 domain. High reactivity was observed in its absence (Flys.ΔHRB), while the reactivity was negligible in the presence of GCN4 (Flys.ΔHRB-GCN). Flys.ΔHRB displayed a similar antibody reactivity profile (high reactivity with 131-2a but not with AM22/D25) as its counterpart that lacks GCN4 but does not contain a deletion of HRB (Flys [14]; S4 Fig). Obviously, the F proteins lacking HRB were not recognized by the antibodies against the 6HB as the 6HB is formed by interaction of HRA with HRB. Treatment of the Flys.ΔHRB-GCN protein samples with limiting amounts of trypsin [14] negatively affected the reactivity of the proteins with the prefusion-specific, but not with the other antibodies (S5 Fig). Our results indicate that Flys.ΔHRB-GCN displays prefusion-like characteristics as it efficiently binds pre- but not postfusion-specific antibodies. The presence of the GCN4 domain and the absence of F protein cleavage contribute to the prefusion-like characteristics of Flys.ΔHRB-GCN.

### Inhibiting the formation of the 6HB does not inhibit presentation of antigenic site I

The prefusion-like characteristics of the non-cleaved, GCN4-containing F protein lacking HRB may be explained by this protein not being able to form the very stable 6HB. To study this possibility, HRB was mutated by the introduction of alanines (Flys.HRBala-GCN; Fig 4), which is expected to interfere with the formation of the 6HB. As a control, cysteines were introduced at the same position in HRB (Flys.HRBcys-GCN). The introduction of these cysteine pairs was previously shown to stabilize the full length protein in the prefusion conformation ([17] and above). The electrophoretic mobility of the resulting F proteins was comparable to that of their HRB-containing counterpart (Flys-GCN). For Flys.HRBcys-GCN an additional higher molecular weight species could be observed (Fig 4B, lane 6). This additional protein band is the only detectable protein species under non-reducing conditions (data not shown) in agreement with the formation of disulfide bridges between the F protein subunits.

**Fig 7:**
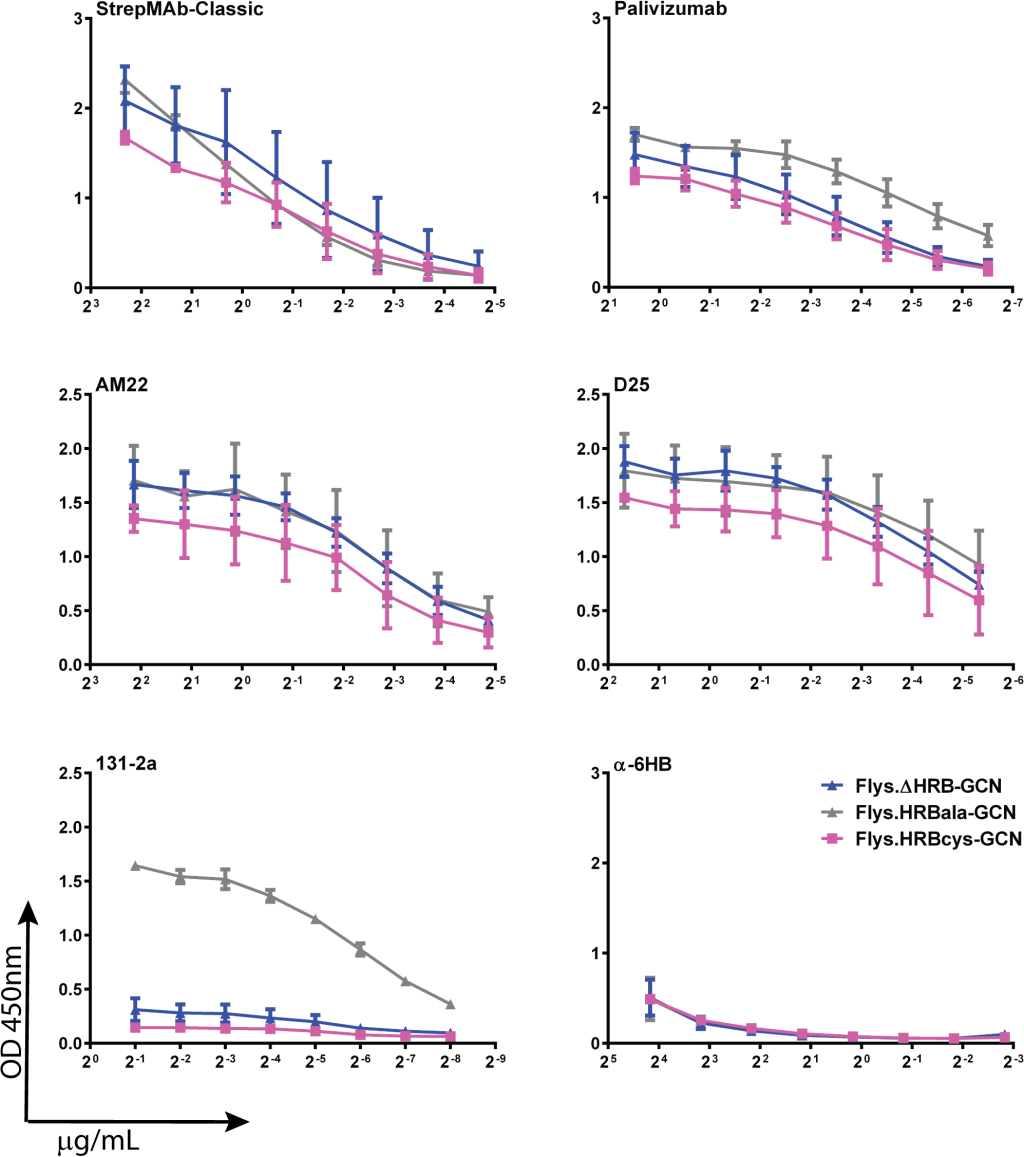
Antigenic analysis of F proteins with mutated HRB. ELISA analysis of purified F proteins Flys.ΔHRB-GCN, Flys.HRBcys-GCN and Flys.HRBala-GCN was performed similarly as described in the legend to Fig 5. Error bars indicate standard deviations (n = 3-5).

Next, the reactivity of the HRB mutant proteins was analyzed with the panel of conformation-specific antibodies (Fig 7 and S6 Fig). Flys.HRBala-GCN and Flys.HRBcys-GCN displayed similar reactivities with Palivizumab, AM22 and D25, which were comparable to those of Flys.ΔHRB-GCN. None of the HRB mutant proteins were able to form the 6HB as demonstrated by their negligible reactivity with α6HB. However, the different F proteins dramatically differed in their reactivity with the postfusion-specific antibody 131-2a (antigenic site I). In contrast to Flys.HRBcys-GCN and Flys.ΔHRB-GCN, Flys.HRBala-GCN was efficiently recognized by 131-2a. From these results, we conclude that mutation of HRB diminishes the formation of the 6HB, but does not necessarily affect the presentation of antigenic site I, as is exemplified by Flys.HRBala-GCN. Of note, Flys.HRBcys-GCN and Flys.ΔHRB-GCN exhibited similar reactivities for all antibodies tested, indicating that these two proteins adopt similar prefusion-like conformations. While the one protein is stabilized by deletion of HRB (Flys.ΔHRB-GCN), the other protein is stabilized by the formation of disulfide bonds in HRB (Flys.HRBcys-GCN).

### Deletion of HRB prevents the presentation of antigenic site I in full-length F protein

**Fig 8:**
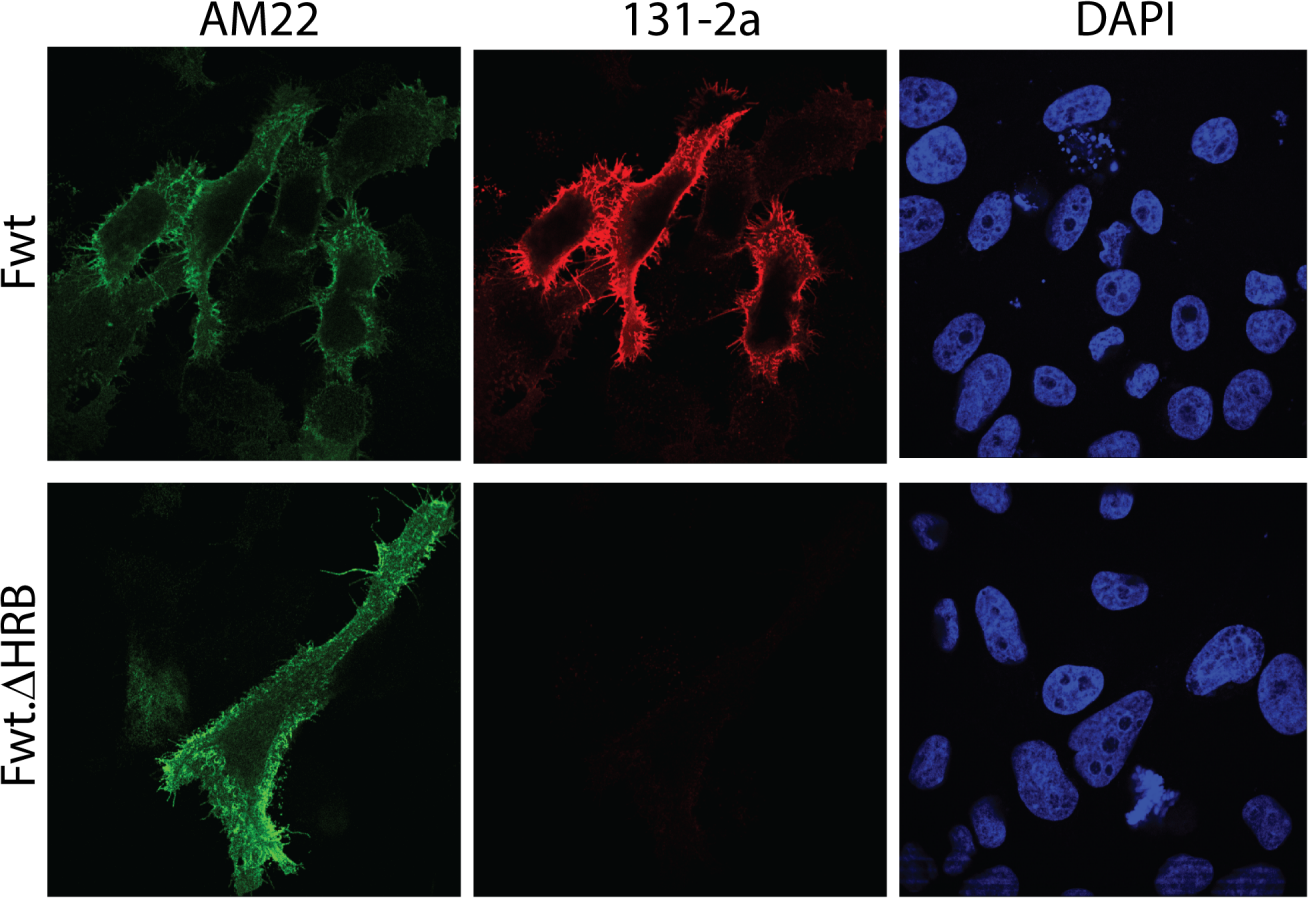
Reactivity of full length RSV F protein lacking HRB with pre- and postfusion-specific antibodies. (A) Cells transfected with expression plasmids for full length wild-type (Fwt) or full length proteins lacking HRB (Fwt.ΔHRB) were fixed and processed for immunofluorescence analysis as described in the Materials and Methods using MAbs AM22 and 131-2a. Nuclei were stained with DAPI.

Finally we tested whether deletion of HRB also prevented the presentation of the postfusion-specific antigenic site I in full length RSV F protein, while maintaining the reactivity with prefusion-specific antibodies. To this end, cells were transfected with plasmids expressing the full length, wild-type F protein or a mutant version thereof, which lacks HRB. Cells expressing wild-type F were recognized by AM22 and 131-2a (Fig 8). Cells expressing the deletion mutant F protein were bound by AM22 to the same extent as cells expressing the wild-type protein. In contrast, negligible binding was observed for 131-2a. These results indicate that epitope I recognized by 131-2a is not exposed on full length F protein lacking HRB, similarly as observed for the non-cleaved, GCN4-containing recombinant soluble F protein lacking HRB or disulfide-bond stabilized F (Fig 3A), suggesting that the full length protein is stabilized in a prefusion-like conformation by deletion of HRB. The results furthermore indicate that in the context of the full length protein preventing cleavage is not critical for preventing the prefusion to postfusion transition.

## Discussion

Recombinant soluble F proteins are potential RSV subunit vaccine candidates, especially those that display high reactivity with highly-neutralizing prefusion-specific antibodies, but not with poorly-neutralizing postfusion-specific antibodies. In the present study we showed that the poorly neutralizing MAb 131-2a recognizing antigenic site I is a postfusion-specific antibody that is not able to recognize F proteins stabilized in the prefusion conformation. Subsequently, we performed a systematic analysis of the reactivity of different recombinant soluble RSV F protein preparations with a panel of conformation-specific antibodies. Non-cleaved, GCN4-extended F protein ectodomain (Flys-GCN) was shown to better display prefusion-specific epitopes compared to cleaved protein lacking GCN4 (Fwt), while it still displayed postfusion-specific antigenic site I and the 6HB epitope(s). In addition, we analyzed in this background the effect of mutations in HRB on the reactivity with the different conformation-specific antibodies. Exposure of antigenic site I could be prevented by deletion of HRB. The resulting F protein displayed a similar antibody recognition profile as prefusion F stabilized via intersubunit disulfide bridges. Absence of cleavage and the C-terminal trimerization domain also contributed to the prefusion-like characteristics of the F proteins. Our results furthermore show that inhibition of 6HB formation *per se* was not sufficient to prevent the conformational change resulting in the display of the postfusion-specific antigenic site I, as expected since 6HB formation follows the conformational change.

Several soluble F protein variants were efficiently recognized by prefusion- as well as postfusion-specific antibodies. Also others reported the reactivity of certain F protein preparations with pre- as well as postfusion-specific antibodies [18]. These observations may be explained by the presence of a mixture of molecules with different conformations in a preparation. Alternatively, it is possible that these F proteins adopt intermediate conformations [23,24] displaying both pre- and postfusion-specific epitopes. Our results also indicate that reactivity of a F protein with a single conformation-specific antibody is not sufficient to draw conclusions about the F protein conformation. Nevertheless, the different antibody recognition profiles of the recombinant soluble RSV F protein preparations analyzed here allow the conclusion that certain F protein modifications (mutation/deletion of HRB or presence of GCN4) are required for maintaining or preventing display of specific epitopes.

The reactivity of the non-cleaved, GCN4-extended RSV F ectodomain with α6HB antibodies indicates that some of molecules form the 6HB, which is characteristic of the postfusion structure. In contrast to the cleaved recombinant soluble F protein, the formation of the 6HB by GCN4-extended non-cleaved F proteins could not be detected after gel electrophoresis followed by Western blot analysis [14], but only by ELISA (this study). Similar results were obtained with proteins that lack the GCN4-trimerization domain ([14] and S4 Fig). We conclude that the 6HB-containing structure formed by the non-cleaved protein is less stable than that of the cleaved protein and therefore not preserved upon SDS-PAGE. The ability of uncleaved paramyxovirus F proteins to adopt a postfusion-like conformation may be a conserved feature as it was also observed for hPIV3 and PIV5 [25,26].

Formation of the 6HB, as detected by ELISA, was prevented by substitution of residues in HRB by alanines. Probably, mutation of HRB abrogates its interaction with HRA. Nevertheless, antigenic site I, which is not available for antibody binding in the prefusion structure, was readily accessible for MAb 131-2a after the introduction of the alanine residues in HRB. This result thus indicates that the formation of the 6HB is not required nor is the driving force for the conformational changes leading to the exposure of antigenic site I. Thus, antigenic site I becomes available for antibody binding prior to the formation of the 6HB. Previously, Russell and coworkers [27] showed that mutations in HRB of paramyxovirus SV5 F protein that destabilized the 6HB structure may result in hyperactive fusion phenotype. Hence, also for the SV5 F protein the 6HB stability alone does not appear to dictate conformational changes in F.

Preventing the formation of the 6HB did not necessarily inhibited the exposure of antigenic site I. The display of antigenic site I was prevented, however, by complete deletion of HRB, in the context of the full length protein or when the non-cleaved soluble protein was extended with a trimerization domain. It is likely that linkage of the GCN4 domain directly to the body of the F molecule is more effective at stabilizing the protein in the prefusion form than linking it to HRB, which is itself not trimerized and therefore may allow triggering. The GCN4-extended F protein lacking HRB showed a very similar antibody recognition profile as that of the recombinant soluble F protein that was stabilized in the prefusion conformation via the introduction of intersubunit disulfide bridges, indicative of these proteins adopting a similar prefusion-like conformation. Higher expression levels were observed, however, for the protein lacking HRB than for Flys.HRBcys-GCN (unpublished data). We hypothesize that the stalk formed by HRB of RSV F is very unstable and has a strong tendency to dissociate resulting in the exposure of antigenic site I. For other paramyxovirus F proteins, residues immediately upstream of HRB have been shown to play a crucial role in the stability of the prefusion conformation [27,28,29]. The exposure of antigenic site I is observed both in the membrane-associated full length protein, but also when the ectodomain of F is extended with a trimerization domain (Flys-GCN) even when mutated so that it is not able to form the 6HB (Flys.HRBala-GCN). However, complete removal of HRB prevented the exposure of antigenic site I.

The current study as well as others [13] indicate that besides the prefusion form also the postfusion form of F is present on infected cells and virus particles. We speculate that the postfusion form of F may function as a decoy antigen during natural infection. This would be in agreement with efficient induction of non-neutralizing F-specific antibodies, presumably targeting postfusion-specific epitopes, upon natural infection [30,31,32]. It will be of interest to study to what extent the presence of postfusion-specific epitopes recognized by poorly neutralizing antibodies such as 131-2a influence the induction of highly neutralizing antibodies against prefusion-specific epitopes. This may particularly be of importance when vaccinating convalescent humans, vaccination of whom may result in boosting of poorly neutralizing antibody responses against postfusion-specific antigenic sites.

## Materials and Methods

### Genes and expression vectors

cDNA clones encoding the RSV F ectodomain (residues 26 to 513; Genbank accession number JX015498.1) of an European isolate [14,33] were synthesized using human-preferred codons by GenScript USA Inc. Each cDNA was cloned into the pCD5 expression vector similarly as described previously [14]. The pCD5 vector had been modified such that the F protein-encoding sequences were cloned in frame downstream of a DNA sequence coding for a CD5 signal peptide and when indicated upstream of sequences encoding an artificial GCN4 isoleucine zipper trimerization motif [34]. The GCN4 domain was fused to the C terminus of F in such a way that the heptad repeat of the GCN is in the same phase as the heptad repeat in HRB [14]. Proteins containing the GCN4 domain were also extended with a LysM domain [35], as the presence of the LysM domain was previously shown to enhance expression and purification of GCN4-containing F protein ectodomains [14]. All F proteins contain a triple Strep-tagII (IBA, Germany) at their C-terminus. cDNA clones encoding the wild-type F protein ectodomain or a F protein ectodomain in which the arginine residues in the two multibasic furin cleavage sites are mutated into lysines (RARR to KAKK and KKRKRR to KKKKKK) have been described before [14]. In addition, pCD5 expression vectors were constructed that encoded F proteins with mutations in HRB. Residues at position 478, 486, 506 and 507 were substituted either by cysteine or alanines or residues 482 to 513 were deleted. These mutations were combined with mutation of the furin cleavage sites and the C-terminal GCN4 trimerization motif and the LysM domain. In addition a pCAGGS mammalian expression vector was constructed that encoded a full length F protein either with or without the above-described cysteine substitutions. Codon-optimized DNA fragments encoding the variable heavy and light chains of antibody Palivizumab [36], D25 [20] and AM22 [19] were synthesized by GenScript USA Inc and cloned in-frame into pCAGGS vectors containing human IgG1 heavy and light constant domains, respectively.

### Expression and purification of recombinant proteins

pCD5 expression vectors containing RSV F ectodomain-encoding sequences were transfected into HEK293T cells as described previously [14]. F proteins were purified using Strep-tactin Sepharose beads according to the manufacturer’s instructions (IBA, Germany). The concentration of Strep-tactin purified protein was determined by using a Nanodrop 1000 spectrophotometer (Isogen Life Sciences). The expression vectors encoding the heavy and light chains were cotransfected at a 1:1 ratio into HEK293T cells similarly as described previously [14]. Antibodies secreted in the cell culture supernatant were bound to protein A sepharose beads (GE Healthcare), after which they were eluted using 0.1 M citric acid pH 3.0. The eluates were immediately neutralized using 1M Tris-HCl pH 8.8. pCAGGS expression vectors encoding the full length F proteins were transfected into HEp-2 cells using lipofectamine 2000 according to the manufacturer’s instructions.

### Gel electrophoresis of recombinant proteins

Purified recombinant F proteins were analyzed by sodium dodecylsulfate (SDS)-polyacrylamide (PA) gel electrophoresis (SDS-PAGE; 10% NuPAGE BisTris, Invitrogen) followed by Western blotting using anti-Strep-tag antibody conjugated with horse radish peroxidase (HRP) (StrepMAB-classic-HRP, IBA). Prior to SDS-PAGE analysis, the samples were resuspended in Laemmli sample buffer (LSB) containing 5% 2-mercaptoethanol (ME; Sigma), and heated at 96°C for 5 minutes.

### Enzyme-linked immunosorbent assay (ELISA) analysis of recombinant soluble F

96-well Nunc maxisorp plates were coated with different F protein preparations (100 ng/well) o/n at 4°C. After blocking (phosphate buffered saline [PBS] with 0.1% Tween-20 v/v and 3% bovine serum albumin w/v) and washing (PBS with 0.05% Tween-20 v/v), the plates were incubated with 2-fold serial dilutions of StrepMab-classic (IBA, start dilution 1:200), Palivizumab (start dilution 1:1000), AM22 (start dilution 1:200), D25 (start dilution 1:200), α6HB [17,22](start dilution 1:100) and 131-2a (Millipore, start dilution 1:2000) using 1mg/ml antibody stocks. After extensive washing, the plates (except for the HRP-conjugated polyclonal goat anti-RSV incubated plates) were incubated with 1:500 dilution of either HRP-conjugated rabbit anti-mouse (Dako), goat anti-human (ITK Southern Biotech) or goat anti-rabbit (Biorad) IgG antibodies for one hour at room temperature. Detection of HRP reactivity was performed using tetramethylbenzidine substrate (BioFX) and an ELISA plate reader (EL-808 from Biotek). All experiments were performed at least three times with independently generated F protein preparations. The mean OD450nm values are depicted in the graphs. One way ANOVA (Graphpad software) was performed using the OD450nm values corresponding to a single dilution of antibody within the linear part of the ELISA curves (S2, S3 and S6 Figs).

### Determination virus neutralizing titers

The RSV neutralizing titers of the monoclonal antibodies were determined by analyzing the reduction of RSV infectivity in the presence of serially diluted monoclonal antibodies in triplicate as described previously [14] using RSV strain Long or GFP-expressing RSV A2 [37]. The 50% inhibition (ng/ml) were determined at the 50% reduction point of the virus control using logarithmic equation derived by plotting dilution versus percentage of infection.

### Immunofluoresence analysis of transfected or infected cells

HEp-2 cells infected with RSV (strain Long) or transfected with full length RSV expression vectors were fixed with 4% formaldehyde in PBS. Cells were washed and incubated with blocking buffer (5% normal goat serum in PBS) after which they were incubated with recombinantly expressed AM22 (1:500 dilution of a 1mg/ml stock), and 131-2a (1:1000 dilution) and with fluorescently-labeled secondary antibodies against human or mouse IgG. After extensive washing with PBS, the samples were mounted on glass slides in FluorSave (Calbiochem). The samples were examined using a confocal fluorescence microscope (Leica TCS SP). For each antibody, pictures were taken with identical settings and laser power. Z stacks were acquired to determine the relative signal intensities of the AM22 and 131-2a staining. Image J was used to process these images and to obtain relative intensities of each stack. The average relative intensity of 12 cells was calculated and subjected to statistical analysis (Student *t* test, Graphpad software; S1 Fig).

### Sandwich ELISA of virus particles

For sandwich ELISA, 96-well Nunc maxisorp plates were coated with 131-2a (100 ng/well) or AM22 (200 ng/well) o/n at 4°C. After blocking (phosphate buffered saline [PBS] with 0.1% Tween-20 v/v and 3% bovine serum albumin w/v) and extensive washing (PBS with 0.05% Tween-20 v/v), the plates were incubated with 2-fold serial dilutions of RSV or RSV that has been heated at 56^°^C for 30 mins. After extensive washing, the plates were incubated with 1:200 dilution of HRP-conjugated goat anti-RSV (Meridian Life Sciences) for one hour at room temperature. Detection of HRP reactivity was performed using tetramethylbenzidine substrate (BioFX) and a ELISA plate reader (EL-808 from Biotek).

## Supporting Information

S1 Fig Reactivity of full length RSV F proteins with pre- and postfusion-specific antibodies. (A) Cells transfected with expression plasmids encoding different full length F proteins (Fwt; wild-type F protein, Fcys; F protein with cysteine pairs in HRB, Fwt.ΔHRB; F protein lacking HRB) were fixed and processed for immunofluorescence analysis as described in the Materials and Methods using MAbs AM22 and 131-2a. Nuclei were stained with DAPI. Bright field images were included as a control. (B) The relative intensities of the AM22 and 131-2a staining for each expression plasmid were depicted as bar charts with error bars representing the standard deviation. Significant differences are indicated (*; *P* values below 0.05). (C) Sandwich ELISA of RSV particles. RSV particles (+RSV) were captured using 131-2a and detected with AM22 or capture using AM22 and detected with 131-2a. As controls the experiment was performed without adding RSV particles (-RSV) or after heating of the particles (+heated RSV).
(TIF)

S2 Fig Antigenic analysis of Fwt and Flys-GCN. The bar graphs depict the OD450nm values corresponding to a single dilution of antibody within the linear part of the ELISA curves shown in Fig 5. Error bars indicate standard deviations. Significant differences are indicated (*; *P* value below 0.05).
(TIF)

S3 Fig Antigenic analysis of Flys-GCN, Flys.ΔHRB-GCN and Flys.ΔHRB. The bar graphs depict the OD450nm values corresponding to a single dilution of antibody within the linear part of the ELISA curves shown in Fig 6. Error bars indicate standard deviations. Significant differences are indicated (*; *P* value below 0.05).
(TIF)

S4 Fig Antigenic analysis of Flys.ΔHRB and Flys. ELISA analysis of purified F proteins Flys.ΔHRB and Flys [14] that lack GCN4 was performed as described in the legend to Fig 5.
(TIF)

S5 Fig Antigenic analysis of Flys.ΔHRB-GCN after proteolysis. ELISA analysis of purified F proteins Flys.ΔHRB-GCN and Flys.ΔHRB-GCN treated with TPCK trypsin (40 μg/ml; [14]) was performed as described in the legend to Fig 5.
(TIF)

S6 Fig Antigenic analysis of Flys.ΔHRB-GCN, Flys.HRBcys-GCN and Flys.HRBala-GCN. The bar graphs depict the OD450nm values corresponding to a single dilution of antibody within the linear part of the ELISA curves shown in Fig 7. Error bars indicate standard deviations. Significant differences are indicated (*; *P* value below 0.05).
(TIF)

## References

[1] Collins PL, Melero JA (2011) Progress in understanding and controlling respiratory syncytial virus: still crazy after all these years. Virus Res 162: 80–99. 10.1016/j.virusres.2011.09.020 21963675 PMC3221877

[2] Del Vecchio A, Ferrara T, Maglione M, Capasso L, Raimondi F (2013) New perspectives in Respiratory Syncitial Virus infection. J Matern Fetal Neonatal Med 26 Suppl 2: 55–59. 10.3109/14767058.2013.831282 24059554

[3] Chang A, Dutch RE (2012) Paramyxovirus fusion and entry: multiple paths to a common end. Viruses 4: 613–636. 10.3390/v4040613 22590688 PMC3347325

[4] Gonzalez-Reyes L, Ruiz-Arguello MB, Garcia-Barreno B, Calder L, Lopez JA, Albar JP, et al (2001) Cleavage of the human respiratory syncytial virus fusion protein at two distinct sites is required for activation of membrane fusion. Proc Natl Acad Sci U S A 98: 9859–9864. 11493675 10.1073/pnas.151098198 PMC55543

[5] Krzyzaniak MA, Zumstein MT, Gerez JA, Picotti P, Helenius A (2013) Host cell entry of respiratory syncytial virus involves macropinocytosis followed by proteolytic activation of the F protein. PLoS Pathog 9: e1003309 10.1371/journal.ppat.1003309 23593008 PMC3623752

[6] Zimmer G, Budz L, Herrler G (2001) Proteolytic activation of respiratory syncytial virus fusion protein. Cleavage at two furin consensus sequences. J Biol Chem 276: 31642–31650. 11418598 10.1074/jbc.M102633200

[7] Schlender J, Zimmer G, Herrler G, Conzelmann KK (2003) Respiratory syncytial virus (RSV) fusion protein subunit F2, not attachment protein G, determines the specificity of RSV infection. J Virol 77: 4609–4616. 12663767 10.1128/JVI.77.8.4609-4616.2003 PMC152164

[8] McLellan JS, Chen M, Leung S, Graepel KW, Du X, Yang Y, et al (2013) Structure of RSV Fusion Glycoprotein Trimer Bound to a Prefusion-Specific Neutralizing Antibody. Science 340: 1113–1117. 10.1126/science.1234914 23618766 PMC4459498

[9] McLellan JS, Yang Y, Graham BS, Kwong PD (2011) Structure of respiratory syncytial virus fusion glycoprotein in the postfusion conformation reveals preservation of neutralizing epitopes. J Virol 85: 7788–7796. 10.1128/JVI.00555-11 21613394 PMC3147929

[10] Swanson KA, Settembre EC, Shaw CA, Dey AK, Rappuoli R, Mandl CW, et al (2011) Structural basis for immunization with postfusion respiratory syncytial virus fusion F glycoprotein (RSV F) to elicit high neutralizing antibody titers. Proc Natl Acad Sci U S A 108: 9619–9624. 10.1073/pnas.1106536108 21586636 PMC3111287

[11] Plattet P, Plemper RK (2013) Envelope protein dynamics in paramyxovirus entry. MBio 4. 10.1128/mBio.00413-13 PMC3705453 23820396

[12] Techaarpornkul S, Barretto N, Peeples ME (2001) Functional analysis of recombinant respiratory syncytial virus deletion mutants lacking the small hydrophobic and/or attachment glycoprotein gene. J Virol 75: 6825–6834. 11435561 10.1128/JVI.75.15.6825-6834.2001 PMC114409

[13] Liljeroos L, Krzyzaniak MA, Helenius A, Butcher SJ (2013) Architecture of respiratory syncytial virus revealed by electron cryotomography. Proc Natl Acad Sci U S A 110: 11133–11138. 10.1073/pnas.1309070110 23776214 PMC3703984

[14] Rigter A, Widjaja I, Versantvoort H, Coenjaerts FE, van Roosmalen M, Leenhouts K, et al (2013) A protective and safe intranasal RSV vaccine based on a recombinant prefusion-like form of the F protein bound to bacterium-like particles. PLoS One 8: e71072 10.1371/journal.pone.0071072 23951084 PMC3741363

[15] Ruiz-Arguello MB, Gonzalez-Reyes L, Calder LJ, Palomo C, Martin D, Saiz MJ, et al (2002) Effect of proteolytic processing at two distinct sites on shape and aggregation of an anchorless fusion protein of human respiratory syncytial virus and fate of the intervening segment. Virology 298: 317–326. 12127793 10.1006/viro.2002.1497

[16] Ruiz-Arguello MB, Martin D, Wharton SA, Calder LJ, Martin SR, Cano O, et al (2004) Thermostability of the human respiratory syncytial virus fusion protein before and after activation: implications for the membrane-fusion mechanism. J Gen Virol 85: 3677–3687. 15557241 10.1099/vir.0.80318-0

[17] Magro M, Mas V, Chappell K, Vazquez M, Cano O, Luque D, et al (2012) Neutralizing antibodies against the preactive form of respiratory syncytial virus fusion protein offer unique possibilities for clinical intervention. Proc Natl Acad Sci U S A 109: 3089–3094. 10.1073/pnas.1115941109 22323598 PMC3286924

[18] McLellan JS, Chen M, Joyce MG, Sastry M, Stewart-Jones GB, Yang Y, et al (2013) Structure-based design of a fusion glycoprotein vaccine for respiratory syncytial virus. Science 342: 592–598. 10.1126/science.1243283 24179220 PMC4461862

[19] Beaumont T, Bakker AQ, Yasuda E (2012) RSV specific binding molecule. US patent application US/2012/0070446 A1.

[20] Kwakkenbos MJ, Diehl SA, Yasuda E, Bakker AQ, van Geelen CM, Lukens MV, et al (2010) Generation of stable monoclonal antibody-producing B cell receptor-positive human memory B cells by genetic programming. Nat Med 16: 123–128. 10.1038/nm.2071 20023635 PMC2861345

[21] Spits H, Beaumont T, Yasuda E, Kwakkenbos MJ (2008) RSV-specific binding molecules and means for producing them. International patent WO/2008/147196.

[22] Palomo C, Mas V, Vazquez M, Cano O, Luque D, Terron MC, et al (2014) Polyclonal and monoclonal antibodies specific for the six-helix bundle of the human respiratory syncytial virus fusion glycoprotein as probes of the protein post-fusion conformation. Virology 460–461: 119–127. 10.1016/j.virol.2014.05.001 25010277

[23] Russell CJ, Jardetzky TS, Lamb RA (2001) Membrane fusion machines of paramyxoviruses: capture of intermediates of fusion. EMBO J 20: 4024–4034. 11483506 10.1093/emboj/20.15.4024 PMC149161

[24] Lamb RA, Jardetzky TS (2007) Structural basis of viral invasion: lessons from paramyxovirus F. Curr Opin Struct Biol 17: 427–436. 17870467 10.1016/j.sbi.2007.08.016 PMC2086805

[25] Yin HS, Paterson RG, Wen X, Lamb RA, Jardetzky TS (2005) Structure of the uncleaved ectodomain of the paramyxovirus (hPIV3) fusion protein. Proc Natl Acad Sci U S A 102: 9288–9293. 15964978 10.1073/pnas.0503989102 PMC1151655

[26] Connolly SA, Leser GP, Yin HS, Jardetzky TS, Lamb RA (2006) Refolding of a paramyxovirus F protein from prefusion to postfusion conformations observed by liposome binding and electron microscopy. Proc Natl Acad Sci U S A 103: 17903–17908. 17093041 10.1073/pnas.0608678103 PMC1635158

[27] Russell CJ, Kantor KL, Jardetzky TS, Lamb RA (2003) A dual-functional paramyxovirus F protein regulatory switch segment: activation and membrane fusion. J Cell Biol 163: 363–374. 14581458 10.1083/jcb.200305130 PMC2173521

[28] Paterson RG, Russell CJ, Lamb RA (2000) Fusion protein of the paramyxovirus SV5: destabilizing and stabilizing mutants of fusion activation. Virology 270: 17–30. 10772976 10.1006/viro.2000.0267

[29] Poor TA, Song AS, Welch BD, Kors CA, Jardetzky TS, Lamb RA (2015) On the stability of parainfluenza virus 5 F proteins. J Virol 89: 3438–3441. 10.1128/JVI.03221-14 25589638 PMC4337539

[30] Anderson LJ, Bingham P, Hierholzer JC (1988) Neutralization of respiratory syncytial virus by individual and mixtures of F and G protein monoclonal antibodies. J Virol 62: 4232–4238. 2459412 10.1128/jvi.62.11.4232-4238.1988 PMC253856

[31] Tsui P, Tornetta MA, Ames RS, Bankosky BC, Griego S, Silverman C, et al (1996) Isolation of a neutralizing human RSV antibody from a dominant, non-neutralizing immune repertoire by epitope-blocked panning. J Immunol 157: 772–780. 8752928

[32] Sakurai H, Williamson RA, Crowe JE, Beeler JA, Poignard P, Bastidas RB, et al (1999) Human antibody responses to mature and immature forms of viral envelope in respiratory syncytial virus infection: significance for subunit vaccines. J Virol 73: 2956–2962. 10074145 10.1128/jvi.73.4.2956-2962.1999 PMC104055

[33] Tan L, Lemey P, Houspie L, Viveen MC, Jansen NJ, van Loon AM, et al (2012) Genetic Variability among Complete Human Respiratory Syncytial Virus Subgroup A Genomes: Bridging Molecular Evolutionary Dynamics and Epidemiology. PLoS One 7: e51439 10.1371/journal.pone.0051439 23236501 PMC3517519

[34] Eckert DM, Kim PS (2001) Design of potent inhibitors of HIV-1 entry from the gp41 N-peptide region. Proc Natl Acad Sci U S A 98: 11187–11192. 11572974 10.1073/pnas.201392898 PMC58705

[35] van Roosmalen ML, Kanninga R, El Khattabi M, Neef J, Audouy S, Bosma T, et al (2006) Mucosal vaccine delivery of antigens tightly bound to an adjuvant particle made from food-grade bacteria. Methods 38: 144–149. 16414272 10.1016/j.ymeth.2005.09.015

[36] Johnson S, Oliver C, Prince GA, Hemming VG, Pfarr DS, Wang SC, et al (1997) Development of a humanized monoclonal antibody (MEDI-493) with potent in vitro and in vivo activity against respiratory syncytial virus. J Infect Dis 176: 1215–1224. 9359721 10.1086/514115

[37] Hallak LK, Spillmann D, Collins PL, Peeples ME (2000) Glycosaminoglycan sulfation requirements for respiratory syncytial virus infection. J Virol 74: 10508–10513. 11044095 10.1128/jvi.74.22.10508-10513.2000 PMC110925

