# Supplementary figures and images for: Recombinant Soluble Respiratory Syncytial Virus F Protein That Lacks Heptad Repeat B, Contains a GCN4 Trimerization Motif and Is Not Cleaved Displays Prefusion-Like Characteristics
Source: PLoS One. 2015 Jun 24;10(6):e0130829. doi: 10.1371/journal.pone.0130829 (PMC4481108; doi:10.1371/journal.pone.0130829)

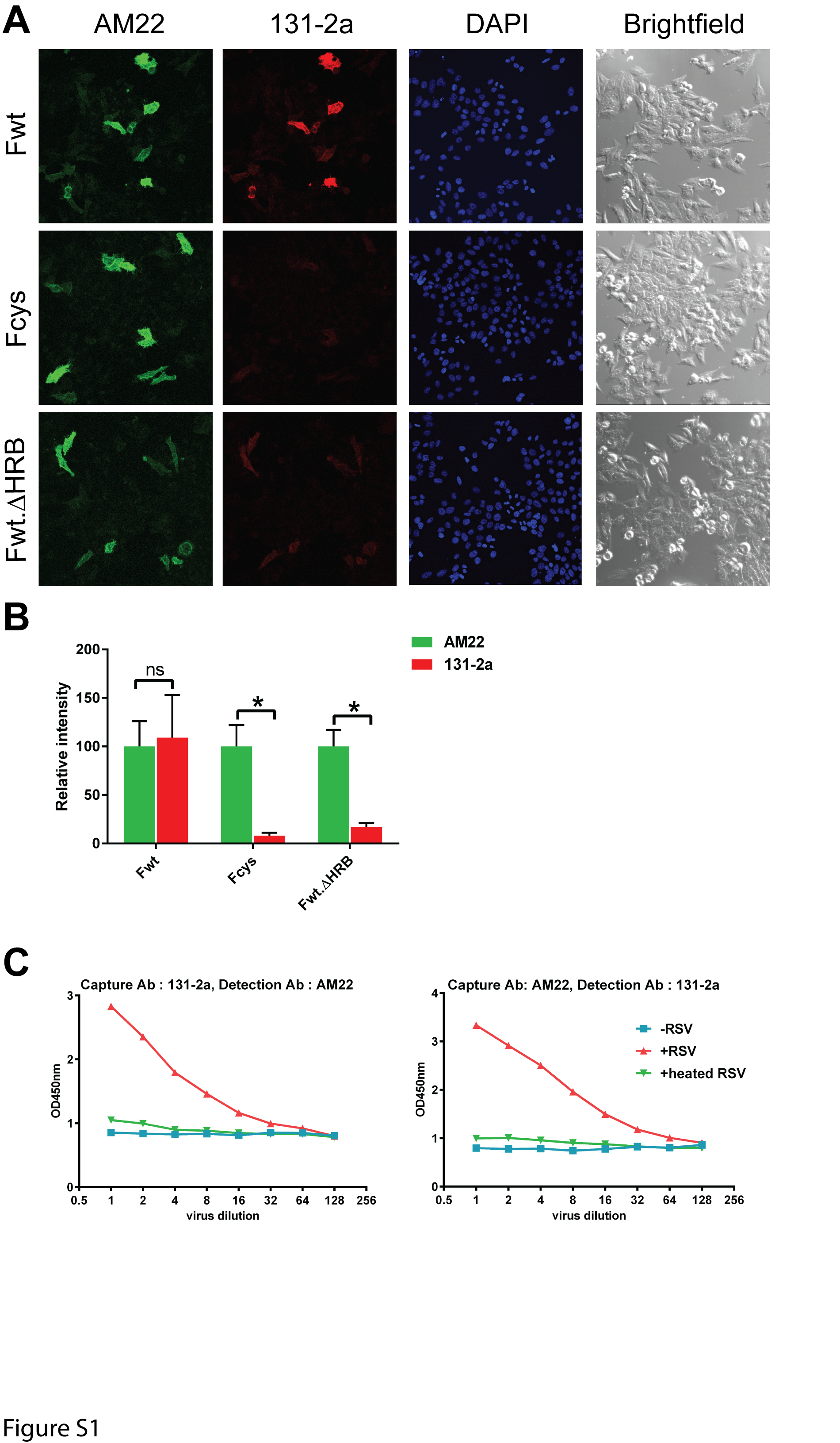

Supplement: S1 Fig — (A) Cells transfected with expression plasmids encoding different full length F proteins (Fwt; wild-type F protein, Fcys; F protein with cysteine pairs in HRB, Fwt.ΔHRB; F protein lacking HRB) were fixed and processed for immunofluorescence analysis as described in the Materials and Methods using MAbs AM22 and 131-2a. Nuclei were stained with DAPI. Bright field images were included as a control. (B) The relative intensities of the AM22 and 131-2a staining for each expression plasmid were depicted as bar charts with error bars representing the standard deviation. Significant differences are indicated (*; P values below 0.05). (C) Sandwich ELISA of RSV particles. RSV particles (+RSV) were captured using 131-2a and detected with AM22 or capture using AM22 and detected with 131-2a. As controls the experiment was performed without adding RSV particles (-RSV) or after heating of the particles (+heated RSV). (TIF) [file pone.0130829.s001.tif]

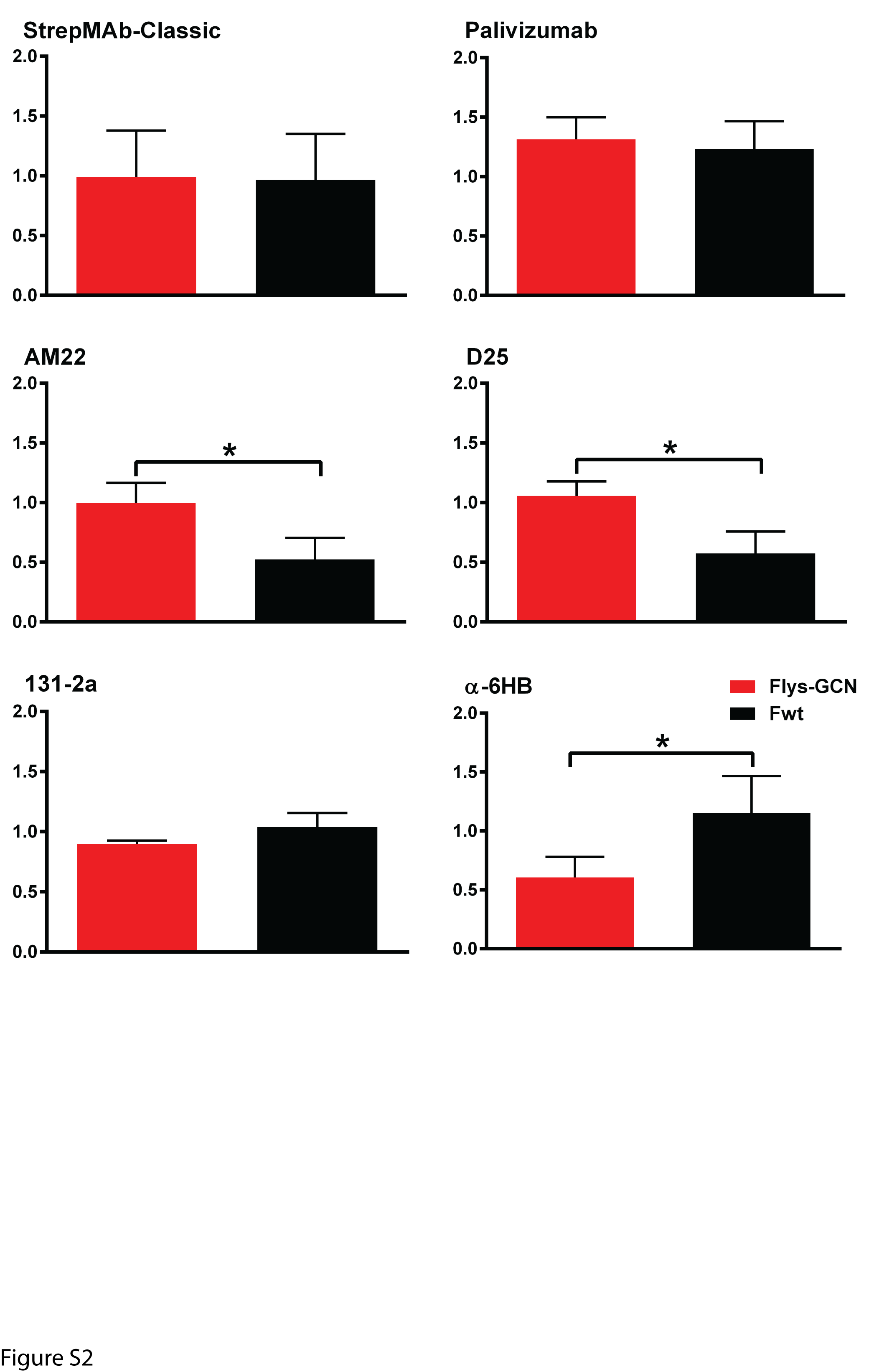

Supplement: S2 Fig — The bar graphs depict the OD450nm values corresponding to a single dilution of antibody within the linear part of the ELISA curves shown in Fig 5. Error bars indicate standard deviations. Significant differences are indicated (*; P value below 0.05). (TIF) [file pone.0130829.s002.tif]

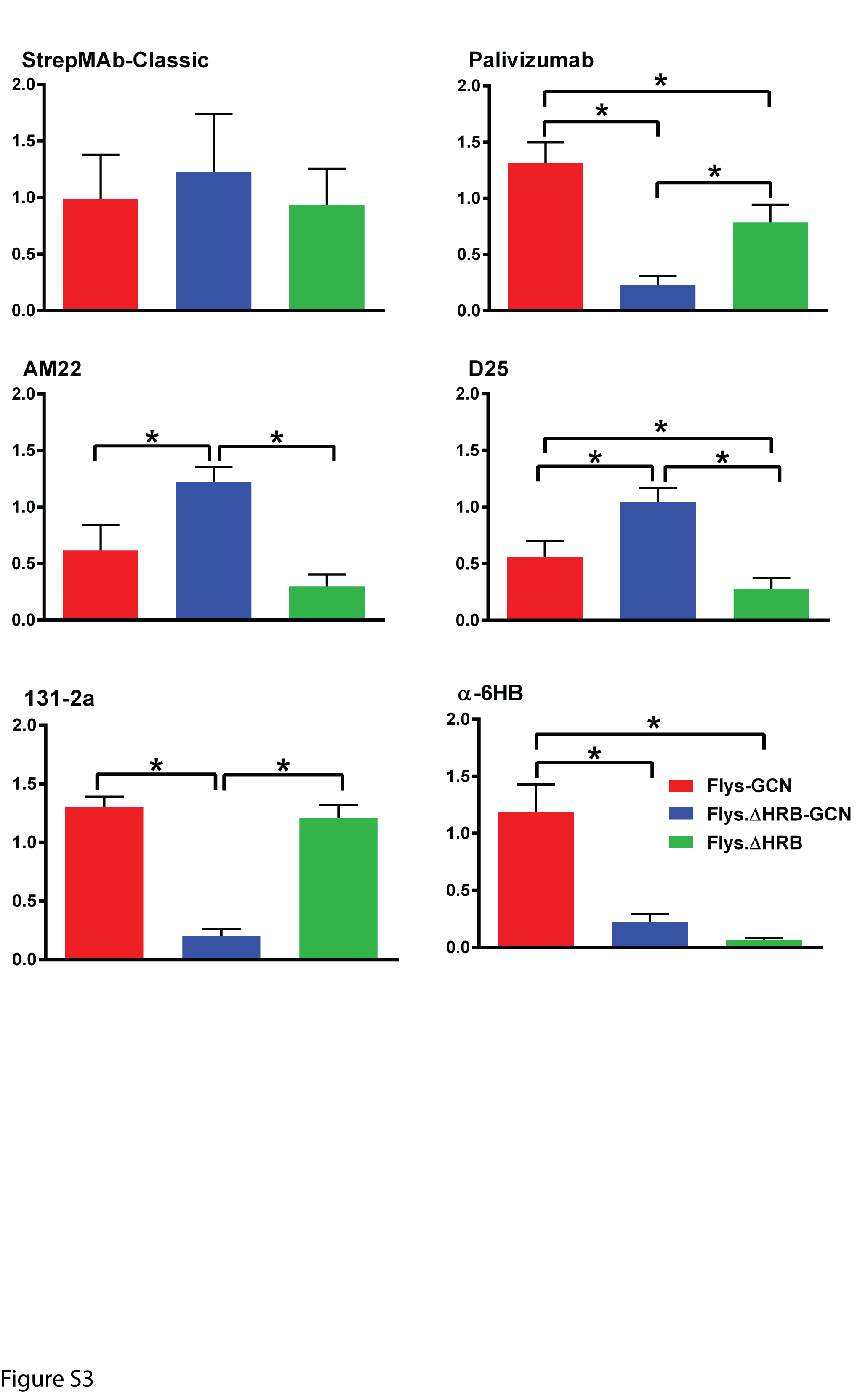

Supplement: S3 Fig — The bar graphs depict the OD450nm values corresponding to a single dilution of antibody within the linear part of the ELISA curves shown in Fig 6. Error bars indicate standard deviations. Significant differences are indicated (*; P value below 0.05). (TIF) [file pone.0130829.s003.tif]

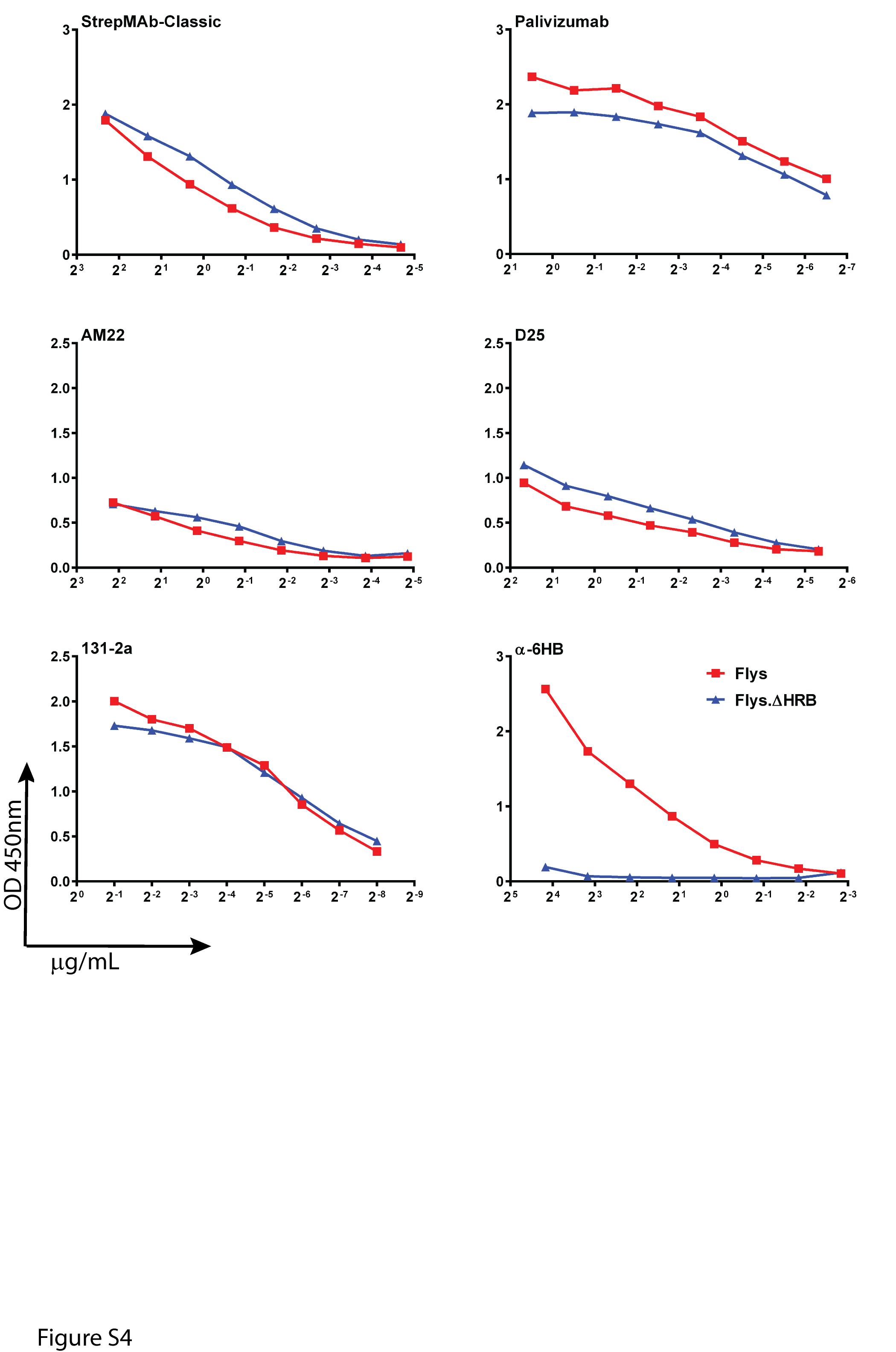

Supplement: S4 Fig — ELISA analysis of purified F proteins Flys.ΔHRB and Flys [14] that lack GCN4 was performed as described in the legend to Fig 5. (TIF) [file pone.0130829.s004.tif]

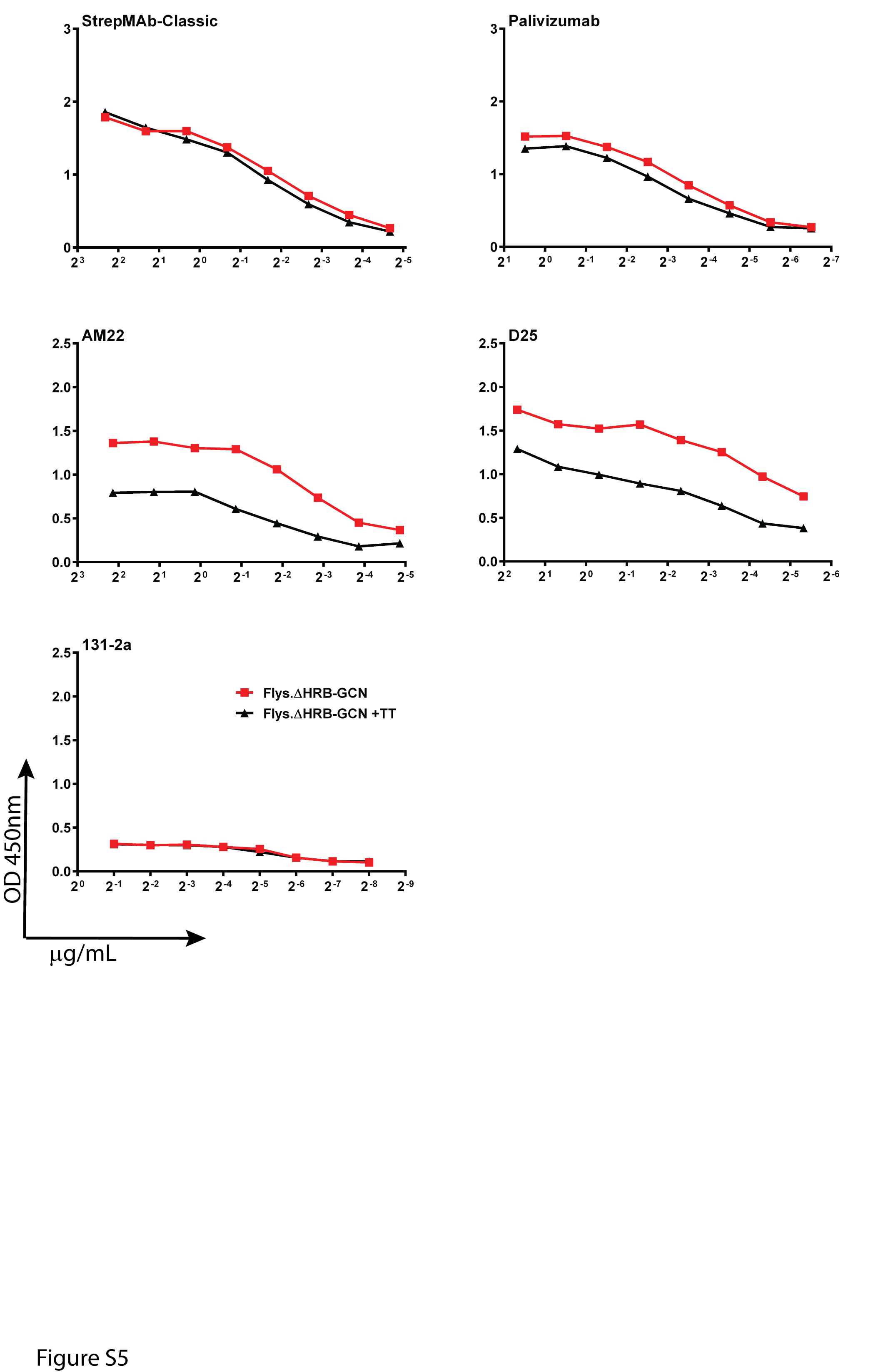

Supplement: S5 Fig — ELISA analysis of purified F proteins Flys.ΔHRB-GCN and Flys.ΔHRB-GCN treated with TPCK trypsin (40 μg/ml; [14]) was performed as described in the legend to Fig 5. (TIF) [file pone.0130829.s005.tif]

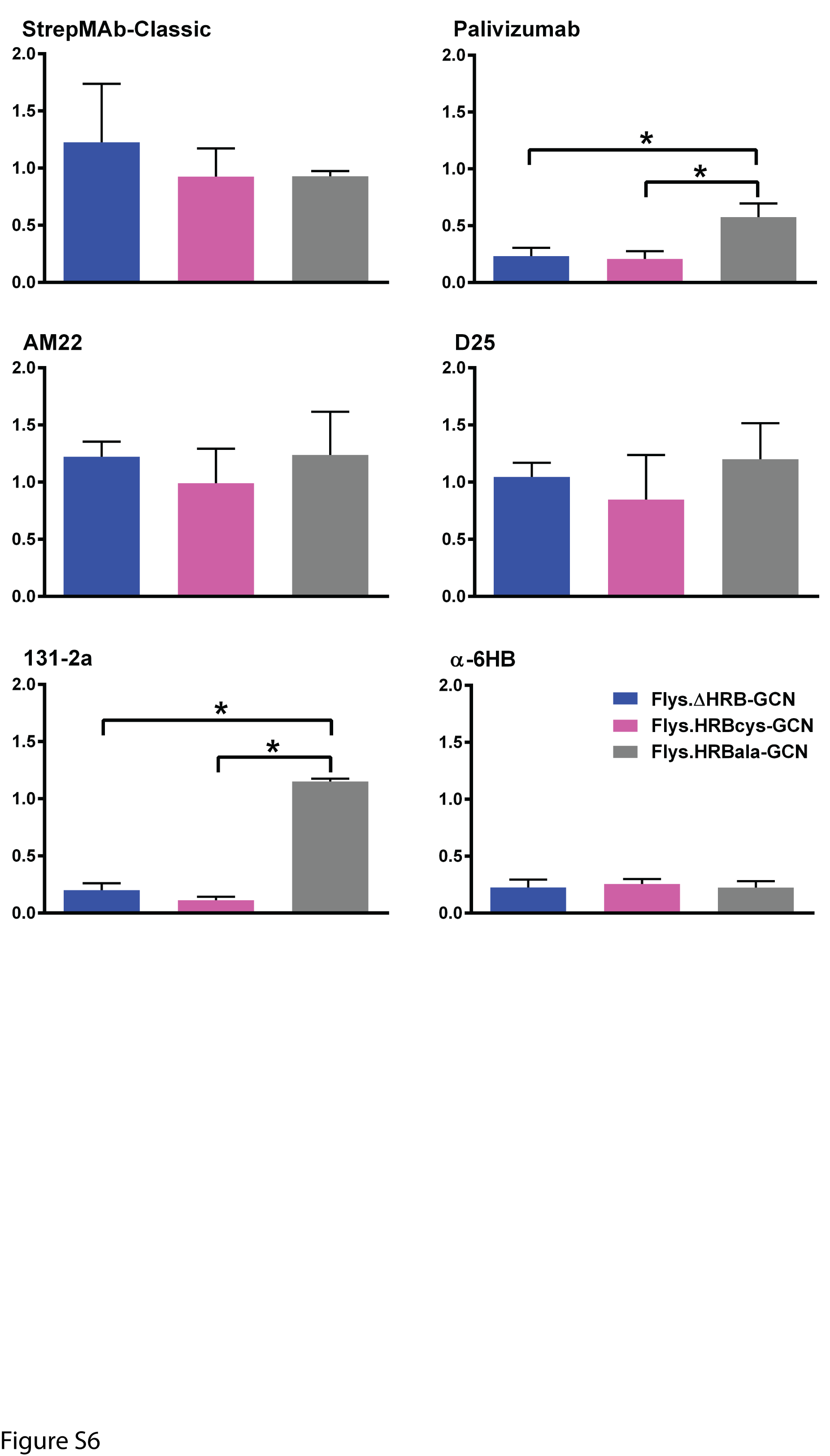

Supplement: S6 Fig — The bar graphs depict the OD450nm values corresponding to a single dilution of antibody within the linear part of the ELISA curves shown in Fig 7. Error bars indicate standard deviations. Significant differences are indicated (*; P value below 0.05). (TIF) [file pone.0130829.s006.tif]
